# Granulomatosis with Polyangiitis Presenting as a Parotid Gland Abscess

**DOI:** 10.1155/2015/865108

**Published:** 2015-03-01

**Authors:** Blenda Dias, Daniela Soares, Patrick Sampaio, Mittermayer Santiago

**Affiliations:** ^1^Service of Rheumatology, Santa Isabel Hospital, Praça Almeida Couto 500, 40050-405 Salvador, BA, Brazil; ^2^Specialized Services in Rheumatology of Bahia, Rua Conde Filho 117, Graça, 40150-150 Salvador, BA, Brazil; ^3^Department of Internal Medicine, Bahiana School of Medicine and Public Health, Avenida Dom João VI 275, Brotas, 40290-000 Salvador, BA, Brazil

## Abstract

Granulomatosis with polyangiitis (GPA) is a small-vessel vasculitis consisting of necrotizing granulomatous lesions in airways and focal necrotizing glomerulonephritis. However, it may affect other sites such as the skin, central nervous system, eyes, heart, gastrointestinal tract, and liver. We describe a rare case of GPA in which the initial manifestation was the involvement of the parotid gland mimicking a pyogenic abscess.

## 1. Introduction

Granulomatosis with polyangiitis (GPA) is a small-vessel vasculitis consisting of necrotizing granulomatous lesions in airways and focal necrotizing glomerulonephritis. However, it may affect other sites such as the skin, central nervous system, eyes, heart, gastrointestinal tract, and liver [1]. Characteristically, there is positivity of autoantibodies to neutrophil cytoplasmic components (cytoplasmic antineutrophil cytoplasmic antibodies, C-ANCA) with specificity to proteinase-3 (PR3).

The aim of this study is to describe a very rare case of GPA in which the initial manifestation was the involvement of the parotid gland mimicking a pyogenic abscess.

## 2. Case Report

**Figure 1:**
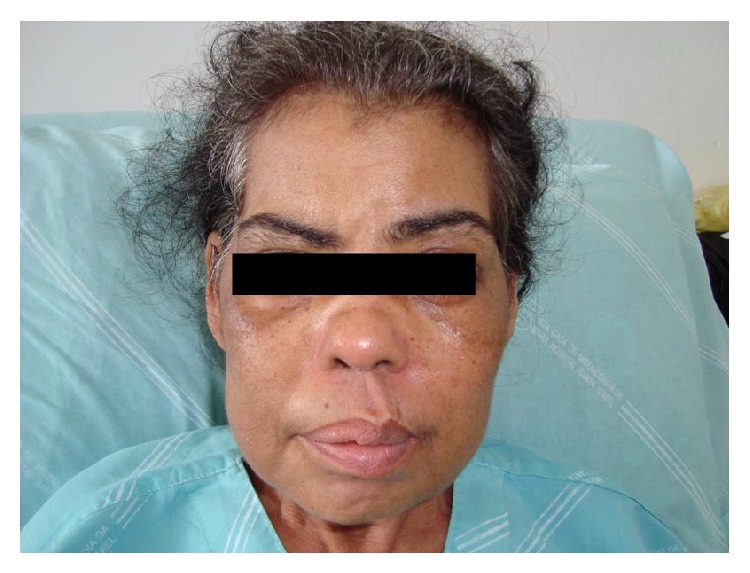
Enlarged right parotid gland and left labial commissural deviation compatible with right peripheral facial nerve paralysis.

A 52-year-old female had a two-month history of painful enlargement of the parotid glands followed by the drainage of purulent secretion from the right parotid that was associated with otalgia, otorrhea and hypoacusia in the right ear, headache, and weight loss (8 kg). She was initially treated with antibiotics (cefepime and clindamycin) in another institution but had no symptomatic improvement. Later, she started presenting fever, cough, sputum, hemoptysis, and dyspnea and was hospitalized in our institution. A physical examination showed her to be in good general condition, afebrile, with a blood pressure of 120/80 mm/Hg. The most remarkable finding was the presence of enlarged and indurated parotid glands that were painful on palpation. There was a left labial commissural deviation compatible with right peripheral facial nerve paralysis (Figure 1). The external auditory canal was edematous with discharge of secretion through a perforation of the middle ear mucosa. Other than these observations, the physical examination was normal. A laboratory workup showed leukocytosis (23,000 cells/mm^3^) without a left shift and an erythrocyte sedimentation rate of 120 mm. Viral serology for hepatitis B and C and HIV was negative. However, C-ANCA by indirect immunofluorescence was positive at 1/80. Initially, thorax radiography was apparently normal but later showed infiltrates and cavities in both lung fields. Computed tomography (CT) showed pleural effusion, an irregular lobular consolidation (measuring 6.3 × 4 cm in its greatest diameter) that was heterogeneous with excavated areas and located in the right upper lobe adjacent to the mediastinal side, and numerous nodular lesions. CT of the face showed an increase in parotids with heterogeneous density and signs of otitis and sinusitis.

Biopsy of the parotid revealed nonspecific chronic inflammation with a few foci of granulomatous tissue mainly represented by histiocytes without vasculitis and search for alcohol acid resistant bacillus (BAAR) was negative. Treatment was initiated with prednisone 1 mg/kg followed by pulse cyclophosphamide (1 g/monthly) for one year and later azathioprine 100 mg/day. This resulted in a significant improvement of the general state and a decrease in the inflammation of the parotid gland. However, she did not recover completely from the facial nerve palsy; there was only a partial improvement in hearing. In a follow-up after four years, she did not present any other systemic manifestation of GPA.

## 3. Discussion

A manifestation of GPA in the parotid gland is unusual. Only few cases have been previously reported [2–11], and to the best of our knowledge the enlargement of the parotid gland mimicking a pyogenic abscess has been described in only two instances [6, 12]. The rarity of salivary gland involvement in GPA, particularly in this atypical presentation, caused some delay in the diagnosis of our patient since the most likely initial hypotheses included a bacterial infection or a neoplastic process. However, the positivity of C-ANCA, the findings from the chest CT, and the presence of a granulomatous process without mycobacterium in the biopsy enabled us to ascertain the diagnosis of GPA. It has been suggested that ANCA in Asian patients with GPA are more likely secondary to antibodies to myeloperoxidase while Caucasian patients have anti-PR3-ANCA even if the vasculitis is not GPA [13]. Unfortunately we could not test the specificity of ANCA in our patient. Besides that this statement has not been tested in the Brazilian population, which is genetically very heterogeneous.

Additionally, a positive response to immunosuppressive medications confirmed this hypothesis. Moreover, the presence of otalgia, hearing loss, and peripheral facial palsy, as it occurred in the present case, is a common feature of GPA involving the parotid gland. Direct injury of the facial nerve by the inflamed parotid gland was the probable reason for the nerve palsy.

Green et al. [5] described one case of parotid gland involvement in GPA and identified a further 41 cases from a comprehensive systematic review of the literature. Among these cases, 60% were males and 40% females with a mean age of 52 years. Curiously, as in our case, the kidneys were less involved than evident in classical GPA. The authors justified this finding on the grounds that a parotid involvement may belong to a more restrict phenotype of the disease. On the other hand, parotid involvement might be an early feature of the disease, which allows prompt treatment and avoids other manifestations of the disease. In our patient, there was no renal involvement in the four-year follow-up.

However, it should be emphasized that the frequency of parotid gland involvement in GPA may be an underestimation. The use of more sensitive diagnostic tools, such as a positron emission tomography (PET) scan, may help define the distribution of the disease and identify the most suitable location for a biopsy [14].

## References

[1] Almouhawis H. A., Leao J. C., Fedele S., Porter S. R. (2013). Wegener's granulomatosis: a review of clinical features and an update in diagnosis and treatment. *Journal of Oral Pathology & Medicine*.

[2] Saravanappa N., Bibas A., Singhal A., Davis J. P. (2000). Unilateral parotid swelling as initial manifestation of Wegener's granulomatosis. *The Journal of Otolaryngology*.

[3] Garcia-Porrua C., Amor-Dorado J. C., Gonzalez-Gay M. A. (2001). Unilateral submandibular swelling as unique presentation of Wegener's granulomatosis. *Rheumatology*.

[4] Chegar B. E., Kelley R. T. (2004). Wegener's granulomatosis presenting as unilateral parotid enlargement. *The Laryngoscope*.

[5] Green I., Szyper-Kravitz M., Shoenfeld Y. (2013). Parotitis as the presenting symptom of wegener's granulomatosis: case report and meta-analysis. *Israel Medical Association Journal*.

[6] Geyer M., Kulamarva G., Davis A. (2009). Wegener's Granulomatosis presenting with an abscess in the parotid gland: a case report. *Journal of Medical Case Reports*.

[7] Ceylan A., Asal K., Çelenk F., Köybaşioğlu A. (2013). Parotid gland involvement as a presenting feature of Wegener's granulomatosis. *Singapore Medical Journal*.

[8] Gassling V., Wiltfang J., Hampe J., Bräsen J. H., Both M., Moosig F. (2010). Salivary gland swelling in Wegener's granulomatosis: a rare cause of a frequent symptom. *The Journal of Rheumatology*.

[9] Frantz M. C., Frank H., von Weyhern C., Kiefer J. (2008). Unspecific parotitis can be the first indication of a developing Wegener's granulomatosis. *European Archives of Oto-Rhino-Laryngology*.

[10] Ullrich S., Heintz H., Gottschlich S., Holl-Ulrich K., Gross W. L., Reinhold-Keller E. (2006). Necrotizing parotitis: an unusual initial manifestation of Wegener's granulomatosis. *Otolaryngology—Head and Neck Surgery*.

[11] Imamoglu M., Bahadir O., Reis A. (2003). Parotid gland involvement as an initial presentation of Wegener's granulomatosis. *Otolaryngology—Head and Neck Surgery*.

[12] Jones G. L., Lukaris A. D., Prabhu H. V., Brown M. J. K. M., Bondeson J. (2005). Wegener's granulomatosis mimicking a parotid abscess. *The Journal of Laryngology and Otology*.

[13] Chen M., Kallenberg C. G. M. (2009). New advances in the pathogenesis of ANCA-associated vasculitides. *Clinical and Experimental Rheumatology*.

[14] Almuhaideb A., Syed R., Iordanidou L., Saad Z., Bomanji J. (2011). Fluorine-18-fluorodeoxyglucose PET/CT rare finding of a unique multiorgan involvement of Wegener's granulomatosis. *British Journal of Radiology*.

